# Association between the Mediterranean Diet and Metabolic Syndrome with Serum Levels of miRNA in Morbid Obesity

**DOI:** 10.3390/nu13020436

**Published:** 2021-01-29

**Authors:** María I. Fontalba-Romero, Soledad López-Enriquez, Ana Lago-Sampedro, Eva Garcia-Escobar, Ricardo L. Pastori, Juan Domínguez-Bendala, Silvia Alvarez-Cubela, Sergio Valdés, Gemma Rojo-Martinez, Eduardo García-Fuentes, María T. Labajos-Manzanares, Sara García-Serrano

**Affiliations:** 1 Unidad de Gestión Clínica de Endocrinología y Nutrición, Hospital Regional Universitario, 29010 Málaga, Spain; mariafontalba82@gmail.com (M.I.F.-R.); anishhh22@gmail.com (A.L.-S.); eyring@gmail.com (E.G.-E.); sergio.valdes@hotmail.es (S.V.); garciasara79@hotmail.com (S.G.-S.); 2 Departamento de Bioquímica Médica, Biología Molecular e Inmunología, Facultad de Medicina, Universidad de Sevilla, 41009 Sevilla, Spain; slenriquez@gmail.com; 4 Instituto de Investigación Biomédica de Málaga-IBIMA, 29010 Málaga, Spain; 5 CIBER de Diabetes y Enfermedades Metabólicas (CIBERDEM), 29009 Málaga, Spain; 6 Diabetes Research Institute, University of Miami Miller School of Medicine, Miami, FL 33136, USA; rpastori@med.miami.edu (R.L.P.); JDominguez2@med.miami.edu (J.D.-B.); salvarez@med.miami.edu (S.A.-C.); 7 Unidad de Gestión Clínica de Aparato Digestivo, Hospital Universitario Virgen de la Victoria, 29010 Málaga, Spain; 8 Faculty of Health Sciences, School of Medicine, University of Málaga, 29071 Málaga, Spain; mtlabajos@uma.es

**Keywords:** Mediterranean diet, metabolic syndrome, miRNA, morbid obesity, type 2 diabetes mellitus

## Abstract

Background: The Mediterranean diet (MD) could be involved in the regulation of different miRNAs related to metabolic syndrome (MS). Methods: We analyzed the serum level of mir-let7a-5p, mir-21, mir-590, mir-107 and mir-192 in patients with morbid obesity and its association with the MD and MS. Results: There is an association between the adherence to MD and higher serum levels of mir-590. Mir-590 was lower in those patients who consumed >2 commercial pastries/week. Mir-let7a was lower in those who consumed ≥1 sweetened drinks, in those who consumed ≥3 pieces of fruit/day and in those who consumed less red than white meat. A lower mir-590 and mir-let7a, and a higher mir-192 level, were found in patients who met the high-density lipoprotein cholesterol (HDL) criterion of MS. A higher mir-192 was found in those patients who met the triglyceride criterion of MS and in those with type 2 diabetes (T2DM). Conclusions: There is an association between specific serum levels of miRNAs and the amount and kind of food intake related to MD. Mir-590 was positively associated with a healthy metabolic profile and type of diet, while mir-192 was positively associated with a worse metabolic profile. These associations could be suggestive of a possible modulation of these miRNAs by food.

## 1. Introduction

The Mediterranean diet (MD) is mainly based on the regular consumption of vegetables, legumes, cereals, fruit and nuts, and the moderate consumption of fish and seafood, balanced by a comparatively limited use of red meat and other meat products, most of them cooked with olive oil as the main source of dietary fat, together with low-to-moderate alcohol consumption [1,2]. This diet has also been associated with a healthy lifestyle (regular physical activity and not smoking) and with a low incidence of cardiovascular disease [3]. The health benefits associated with the MD have been subsequently associated not only with the prevention of cardiovascular disease but also with the prevention of type 2 diabetes (T2DM), obesity, metabolic syndrome, cancer and neurodegenerative diseases [4,5]. However, due to the current changes in lifestyle, which trend towards a sedentary lifestyle and the increased consumption of ultra-processed products and high caloric content, this healthy lifestyle associated with the MD is being worryingly extinguished [6]. These changes in lifestyle are causing a significant increase in obesity and associated metabolic diseases in populations, such as the decrease in high-density lipoprotein cholesterol (HDL) levels, elevated triglyceride levels, increased blood pressure (BP) and hyperglycemia [7,8]. The beneficial effects provided by the MD to human health reside mainly in its bioactive nutrients and components [9,10,11,12]. However, the molecular mechanisms through which MD produces these benefits remain unclear. Several studies show that the diet could be a factor involved in the evolution and risk of cardiovascular disease [13].

MiRNAs could be used as biomarkers and as a possible pharmacological target for cardiovascular diseases. We hypothesized that miRNAs may influence such effects. MiRNAs are involved in epigenetic control and, practically since their discovery, these small molecules of RNA were proposed as a new means of communication between different metabolic tissues [14,15]. Several studies show that specific nutritional factors could regulate the miRNA expression in different cells/tissues related to obesity, T2DM and associated diseases, with consequent effects on the metabolism [16,17,18,19,20,21]. Among these nutritional factors that can affect to miRNAs expression are the composition in fatty acids, vitamins, minor compounds present in fruits and vegetables, minerals, fiber, etc. [13]. MiRNAs also play an outstanding role in the field of biomarkers for many diseases as well as being therapeutic tools [22].

Food frequency questionnaires are tools that allow us to assess the type of diet and the quantity of food consumed. The method closest to the gold standard for evaluating food consumption involves the use of questionnaires that evaluate multiple foods over more or less long periods of time [23,24]. The use of these questionnaires requires time consuming, both in terms of data collection and analysis. The Mediterranean Diet Adherence Screener (MEDAS) is a short food frequency questionnaire to evaluate the adherence to the MD in a simple questionnaire of 14 items. MEDAS is obtained in a single visit and it has been adapted from a more extensive questionnaire to facilitate its use [25]. The validity of this questionnaire has been previously analyzed in different countries [26,27,28,29,30,31,32,33] and in a simultaneous and comparative cross-national validation study [34]. In all of them, the MEDAS questionnaire has been a reliable and valid tool to screen adherence to the MD.

Therefore, the aim of the study was to evaluate whether there is an association between adherence to the MD in a group of patients with morbid obesity and several serum levels of miRNA associated with obesity and T2DM. Although this type of patient is not a representative population of a very healthy lifestyle, among them there are always patients whose diet allows them to be considered as adherents to the MD, and this could be associated with a better metabolic profile. In this relationship between metabolic syndrome/obesity/T2DM and adherence to MD, miRNAs may be playing a relevant role.

## 2. Materials and Methods

### 2.1. Participants

Fifty-eight patients with morbid obesity (body mass index (BMI) ≥40 kg/m^2^) were included from the Regional University Hospital of Malaga (Spain) [35]. This research was carried out in accordance with the Declaration of Helsinki of the Word Medical Association. Written informed consent was obtained from all the participants. The study was approved by the Ethics Committee of the Provincial Research of Malaga (Malaga, Spain) (PI18/01165).

### 2.2. Clinical Variables and Information about Lifestyle

The information was obtained by means of a single visit with a structured questionnaire, which was filled out by a nurse and was followed by a physical examination. Weight and height determinations and waist and hip perimeters were measured with standardized methods and calibrated scales [36]. Blood pressure was recorded with a blood pressure determination device (Hem-703C, Omron, Barcelona, Spain) with the participant seated and after 5 min of rest. Two readings were obtained and their mean was used in the analyses [37]. Metabolic syndrome was classified according to the International Diabetes Federation (IDF) criteria [38].

Adherence to the MD was determined by means of MEDAS, a scored food frequency questionnaire that was previously validated [25,26,27,28,29,30,31,32,33]. It is a 14 item questionnaire of food consumption to evaluate the amount of 12 main components consumed and two food habits related to the MD. Each of the 14 items is scored with 1 point or 0 points, depending on whether participants are adherent to each component of the MD (1 point) or not (0 points) Table 1. Those subjects with a score ≥9 were considered as adherent to the MD (adhMD) and those subjects with a score <9 were considered not to be adherent to the MD (non-adhMD) [25]. Although the food frequency questionnaire was obtained in a single visit, the information obtained is based on intake over the last month and it is representative of the lifestyle and food intake of each subjects.

### 2.3. Biochemical Determinations

After 10 h fasting, a venous blood sample was drawn and an oral glucose overload test (75 g of glucose, 2 h) was performed on each subject. Samples were immediately centrifuged and serum samples were stored at −80 °C for later analysis. Serum glucose, triglycerides and cholesterol were measured enzymatically and HDL cholesterol by direct method on an Architect C8000 Analyzer (Abbott Laboratories SA, Madrid, Spain). Serum insulin was measured by immunochemiluminescence on an Architect I8000 Analyzer (Abbott Laboratories SA). Low-density lipoprotein (LDL) cholesterol was estimated by the Friedewald formula. The homeostatic model assessment insulin resistance index (HOMA-IR) was calculated by the formula: (serum glucose (mmol/L) × serum insulin (mU/L))/22.5.

### 2.4. MiRNA Determinations

We selected different candidate miRNAs based on a significant differential expression level according to the presence of T2DM in a previous screening (data not published). The screening was performed in 32 serum samples of patients with morbid obesity with similar biochemical and anthropometric characteristics to the population of our study (data not shown). A total of 742 microRNAs were screened with the LNA™ Universal miRCURY microRNA RT PCR, 4× Human Panel I + II in 384 well plates PCR (Exiqon A/S Vedbaek, Denmark). Five miRNAs were finally included according to this criterion: mir-let7a-5p, mir-21, mir-590, mir-107 and mir-192.

The extraction of miRNAs from serum samples was performed by means of automated methods in a Maxwell 16 de Promega (serial number 23627808) with the Maxwell^®^ 16 miRNA Tissue Kit (Promega Biotech Ibérica S.L., Madrid, Spain) and converted to microcDNA by reverse transcription with the Universal cDNA Synthesis Kit (Exiqon A/S Vedbaek, Denmark) following the manufacturer’s recommendations for each kit. Measurements of miRNA expression levels were performed by qPCR real time in 384 plates in a Light Cycler 480 (Roche Diagnostics, S.L, Barcelona, Spain) at the Genomics platform of the Biomedical Research Institute of Malaga (IBIMA). The master mix was prepared following the guidelines of Exiqon with GoTaq(R) qPCR Master Mix (Promega Biotech Ibérica S.L., Madrid, Spain) and the specific LNA™ PCR primer sets (Exiqon A/S Vedbaek, Denmark). The analysis of miRNAs was performed using the delta Ct method. Mir-484 was selected as the normalizer miRNA after an analysis with RefFinder (https://www.heartcure.com.au/reffinder/?type=reference), found it to be the most stable miRNA from the previous screening, mentioned above, performed on subjects with morbid obesity (data not shown). For data normalization, negative controls and a specific calibrator in all plates for interplate normalization were included. Those determinations of miRNAs with Ct ≥ 38 were considered under the limit of detection and were taken out of the analysis.

### 2.5. Statistical Analysis

The sample size was calculated from the expression of mir-590 in the serum of patients with morbid obesity from a preliminary study. We estimated that a sample size of 8 patients would have 80% power to detect a difference between means of fold change of 0.35, with a standard deviation of 0.24 and type 1 error-alpha of 0.05. Statistical analysis was performed using the SPSS software (IBM SPSS Statistics, SPSS, Chicago, IL, USA). Comparison between variables of the different groups was made with the Mann–Whitney test, lineal and logistic regressions. Analysis by lineal regression for the dependent variable miRNA expression was performed with adherence to the MD, age, sex and body mass index as covariates. Analysis by logistic regression for the dependent variable adherence to the MD and metabolic syndrome was performed with age, sex and body mass index as covariates. Chi-square test was used to evaluate the degree of association between categorical variables. Values were considered to be statistically significant when *p* ≤ 0.05.

## 3. Results

### 3.1. Anthropometric and Biochemical Characteristics

Table 2 summarizes the characteristics of patients with morbid obesity classified as adhMD (n = 8) and non-adhMD (n = 50). No significant differences were found between both groups. Only diastolic blood pressure (DBP) values were significantly greater in the non-adhMD group.

### 3.2. Serum miRNA Levels Regarding Adherence to the MD

First, we analyzed whether the expression of the miRNAs correlated with one another. We found that mir-590 significantly correlated with mir-let7a (r = 0.361, *p* = 0.006), and mir-192 with mir-107 (r = 364, *p* = 0.005).

**Figure 1:**
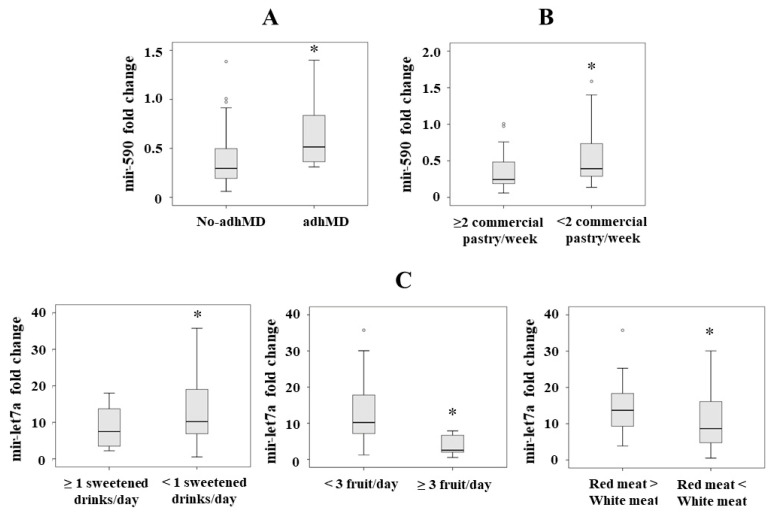
(**A**) Mir-590 serum levels in patients with morbid obesity classified as not adherent (no-adhMD) and adherent to the Mediterranean diet (adhMD). (**B**) Mir-590 serum levels in patients with morbid obesity according the commercial pastries/week consumption. (**C**) Mir-let7a serum levels in patients with morbid obesity according to type of meat, fruit and sweetened drinks consumption. Results are represented as median. * *p* < 0.05.

We hypothesized that there would be significant differences in the levels of the serum miRNAs selected in our study (mir-590, mir-107, mir-let7a, mir-21 and mir-192) regarding adherence or non-adherence to MD. We only observed significant differences in mir-590 serum levels (*p* = 0.042), with a higher level in those patients with adherence to the MD (Figure 1A). Next, we analyzed the association between these miRNAs, as a dependent variable in a lineal regression analysis, and adherence to MD adjusted by sex, age and body mass index. We only observed significant the association between mir-590 and adherence to the MD (*p* = 0.040, B = 0.275).

### 3.3. Serum miRNA Levels with Respect to Individual MEDAS Items 

Next, we hypothesized that significant differences would be observed in the levels of those serum miRNAs with respect to each of the 14 individual MEDAS items. These results are summarized as follow.

#### 3.3.1. Mir-590

We found significantly lower levels in those patients who consumed >2 commercial pastries/week (Item 11) (*p* = 0.040) (Figure 1B). By lineal regression analysis, we only observed significant the association between mir-590 and item 11 (consumption of commercial pastries/week) (*p* = 0.032, B = 0.286).

#### 3.3.2. Mir-Let7a

We found lower serum mir-let7a levels in those patients who consumed ≥1 sweetened drinks (*p* = 0.043) (Item 7), in those who consumed ≥3 pieces of fruit/day (*p* = 0.045) (Item 4), and in those who consumed less red than white meat (*p* = 0.048) (Item 13) (Figure 1C). By lineal regression analysis, we observed significant associations between mir-let7a and item 7 (consumption of sweetened drinks) (*p* = 0.022, B = 0.322), mir-let7a and item 4 (consumption of pieces of fruit/day) (*p* = 0.027, B = −0.293) and mir-let7a and item 13 (consumption of less red than white meat) (*p* = 0.049, B = −0.255).

### 3.4. Relationship between Adherence to the MD and Metabolic Syndrome

We also analyzed whether there was an association between adherence to the MD, according to the MEDAS questionnaire, and the presence of metabolic syndrome and its criteria. There is no significant association between adherence to the MD and the presence of metabolic syndrome and its criteria. Since all patients with morbid obesity met the waist circumference criterion, we could not perform statistical analyses for it.

### 3.5. Serum miRNA Levels with Respect to Metabolic Syndrome

After observing the relationship between adherence to the MD and metabolic syndrome, we analyzed whether there were significant differences in the levels of the serum miRNA studied according to the presence of metabolic syndrome and/or its criteria. We did not observe any significant differences in the serum miRNA levels regarding whether they have metabolic syndrome or not (data not shown).

**Figure 2:**
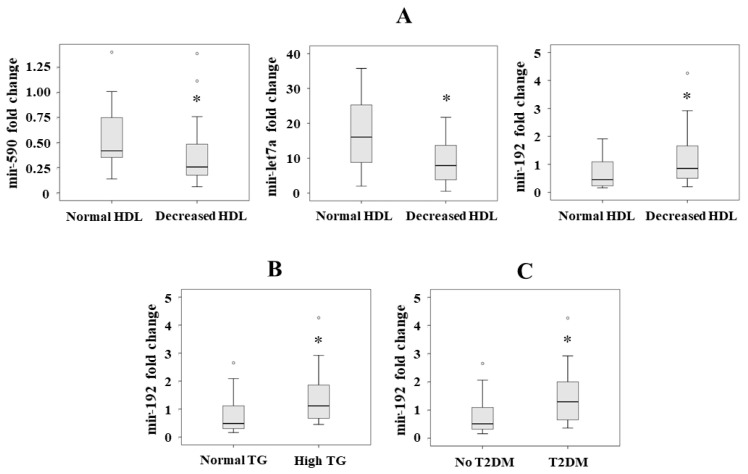
Significant serum levels of miRNAs according to the presence of the different criteria of metabolic syndrome: (**A**) mir-590, mir-let7a and mir-192 levels according to HDL criterion, (**B**) mir-195 levels according to triglyceride (TG) criterion, and (**C**) mir-192 levels according to the presence of T2DM. Results are represented as median. * *p* < 0.05.

Although we have not found differences in serum miRNA levels according to the presence of metabolic syndrome, some significant differences were found according to the criteria of metabolic syndrome. After different logistic regression analyses with the criteria of metabolic syndrome as dependent variables, adjusted by sex, age and body mass index, we only observed significant differences in the following miRNA levels: lower mir-590 (*p* = 0.032, OR = 0.112, 95% CI = 0.015–0.829) and mir-let7a (*p* = 0.003, OR = 0.867, 95% CI = 0.789–0.953), and higher mir-192 (*p* = 0.048, OR = 2.649, 95% CI = 1.010–6.950) levels in patients who met the HDL criterion of metabolic syndrome (Figure 2A). In addition, a higher mir-192 level was found in those patients who met the triglyceride criterion (*p* = 0.017, OR = 2.742, 95% CI = 1.194–6.297) of metabolic syndrome (Figure 2B). We did not find any statistically significant differences in the serum miRNA levels analyzed with respect to hypertension and glucose criteria (data not shown).

### 3.6. Association between Serum miRNA Levels and the Components of the Metabolic Syndrome

We analyzed the association between the miRNAs and the presence of T2DM as a dependent variable in a forward stepwise logistic regression analysis, adjusted by sex, age and body mass index. We observed that mir-192 was the only associated variable (*p* = 0.013, OR = 2.643, 95% CI = 1.223–5.709) (Figure 2C).

## 4. Discussion

In this study, we have found a significant association between adherence to the MD and a higher level of mir-590. We observed that this miRNA, together with others also analyzed here, is associated with the amount and kind of food intake, mainly fruit, commercial pastries, sweetened drinks and red or white meat. The lipid profile related to metabolic syndrome was associated with the miRNAs studied: the HDL criterion was associated with lower levels of mir-590 and mir-let7a, and with higher serum levels of mir-192. We also observed that the triglyceride criterion was associated with higher serum levels of mir-192. High serum levels of mir-192 were also associated with the presence of T2DM.

Different studies have revealed that individual bioactive nutrients are responsible for the cardioprotective effects of some dietary plans (e.g., the MD). However, the direct mechanisms of action are still not fully understood. Bioactive dietary components could have the ability to change miRNA expression, thereby modulating important pathways involved in metabolism [20,21,39,40]. MiRNAs are involved in the expression of a widespread range of proteins and their consequent effect on metabolic functions and the maintenance of oxidative stress [41]. Therefore, it seems reasonable to assume that some bioactive compounds present in different foods may modulate the development and progression of certain diseases through miRNA. In this scenario, we have found how different miRNAs are associated with adherence to the MD and/or with some of the foods that are considered in the questionnaire of adherence to the MD in patients with morbid obesity. The main miRNA that we found associated with adherence to the MD and metabolic syndrome is mir-590. There was an association between higher mir-590 levels and adherence to the MD, as well as with a lower consumption of pastry. With respect to the metabolic syndrome criteria, higher levels of mir-590 were associated with HDL in the normal range. These results suggest that higher levels of this miRNA are associated with healthy eating/diet habits, and with a better metabolic profile. To date, we have not found any study on a possible relationship between mir-590 and the modulation/association with HDL levels. However, there are studies that have shown that the overexpression of mir-590 inhibits the atherosclerotic lesion in HFD-induced apoE^−/−^ mice, preserves cell proliferation and inhibits apoptosis of oxidized low-density lipoprotein-treated endothelial cells [17]. This effect would be by means of inhibition of the TLR4/NF-κB pathway. Another study has provided evidence that mir-590 could be related to T2DM in patients with Blood Stasis Syndrome, which has been associated with many cardiovascular diseases such as hypertension and T2DM [42]. Another study also demonstrated how mir-590 suppressed the activity of the lactate dehydrogenase A (LDHA), an enzyme whose up-regulation is found in human and rodent T2DM models [43]. Regarding the modulation of mir-590 by food, we only found one study in which curcumin protects the endothelium by means of inhibiting the expression of CD40 and oxidative stress in a mir-590-3p dependent pathway [44]. Overall, our results, together with other studies, indicate that mir-590 could be involved in both lipid and glucose metabolism and modulated in response to specific foods or dietary patterns.

Regarding mir-192, our study shows a consistent pattern: a lower serum level of mir-192 is associated with higher HDL and triglyceride levels in the normal range. However, our results seem to disagree with other studies regarding this miRNA, where mir-192-5p was decreased both in livers with steatosis and cells with lipid accumulation [45]. In addition, another study indicated that cellular TG increased after the inhibition of mir-192-5p, and this effect could be blocked by the transfection of SCD-1 [46]. SCD-1 is an enzyme that catalyzes the desaturation of fatty acyl-CoA substrates and seems to be negatively associated with the insulin resistance state in visceral adipose tissue [47]. However, those studies were performed in different tissues but not in serum, so our results could have a different explanation. Mir-192 shows a repressive effect on the expression of genes involved in lipid metabolism, such as the very low-density lipoprotein receptor (VLDLR) [48]. Thus, low levels of mir-192 would produce an increase in VLDLR and serum triglyceride levels would remain at normal levels, with a possible increase in other tissues. On the other hand, we also found a lower serum level of mir-192 in those patients who did not meet the criteria for T2DM. This means that low levels of mir-192 are associated with low glucose levels. In line with this, a study performed in prediabetic subjects identified high levels of circulating mir-192-5p as a biomarker of prediabetes [49], being involved in the modulation of different pathways related to T2DM [50]. Together, our results seem to suggest that low levels of this miRNA could have healthy effects on the metabolism. With regard to its modulation by diet, a study has shown a modulation of mir-192 by polyunsaturated fatty acids from diet. Docosahexaenoic acid can up-regulate mir-192 levels in enterocyte-like Caco-2 cells [48].

Regarding mir-let-7a, we would find a situation that is opposite to mir-192. Low levels of this miRNA are found in those patients with a worse metabolic situation (consumption of one or more than sweetened drinks per day), and in those patients who meet the criteria of HDL for metabolic syndrome classification. However, we also found lower mir-let-7a levels in subjects with a higher consumption of white meat compared to the consumption of red meat, and in those with a higher consumption of fruit. Accordingly, some studies show an upregulation of mir-let-7a in the pancreatic ductal by quercetin. This molecule is a kind of polyphenol used as nutritional supplements, which is mainly in fruit [51,52]. The upregulation of the mir-let-7 family function is mainly associated with both prognosis and therapy for precision medicine in cancer. It is known that this pathology is associated with obesity, but that it is mainly associated with colorectal cancer [53].

This study has several limitations. These miRNAs were selected from a previous screening, and were predicted to be potential candidates for differential regulation regarding metabolic criteria. However, other miRNA could be also associated with the type of diet. We only analyzed a few variables related to the diet according to the MEDAS questionnaire, rather than particular foods, which could provide other results. Our data cannot be extrapolated to non-obese patients. Patients with morbid obesity have peculiar nutritional characteristics in terms of type and quantity of food intake. In addition, due to the small size of groups, we consider these results as a preliminary study.

## 5. Conclusions

We have identified miRNAs associated with the different criteria of metabolic syndrome and with the amount and type of diet, mainly fruit, sweetened drinks, commercial pastries and red or white meat. Although there is a genetic predisposition to the pathologies associated with metabolic syndrome, lifestyle and diet could also be involved in its development. Some of these miRNAs, such as mir-590, were positively associated with a better metabolic profile and type of diet, while other miRNAs, such as mir-192, were positively associated with a worse metabolic profile. These suggest a possible modulation of these miRNAs by food. However, the direct mechanisms of action are still not fully understood. Other in-vitro and in-vivo studies are needed to establish the molecular mechanisms behind these associations. Dietary components could have the ability to change miRNA expression, thereby modulating important pathways involved in lipid and glucose metabolism.

## Figures and Tables

**Table 1:** Mediterranean Diet Adherence Screener (MEDAS) questionnaire.

1. Do you use olive oil as the principal source of fat for cooking?	1 point given: yes
2. How much olive oil do you consume per day (including that used in frying, salads, meals eaten away from home, etc.)?	1 point given if ≥4 Tbsp ^1^
3. How many servings of vegetables do you consume per day? Count garnish and side servings as 1/2 point; a full serving is 200 g.	1 point given if ≥2
4. How many pieces of fruit (including fresh-squeezed juice) do you consume per day?	1 point given if ≥3
5. How many servings of red meat, hamburgers, or sausages do you consume per day? A full serving is 100–150 g.	1 point given if <1
6. How many servings (12 g) of butter, margarine, or cream do you consume per day?	1 point given if <1
7. How many carbonated and/or sugar-sweetened beverages do you consume per day?	1 point given if <1 ^2^
8. Do you drink wine? How much do you consume per week?	1 point given if ≥7 cups
9. How many servings (150 g) of pulses do you consume per week?	1 point given if ≥3
10. How many servings of fish/seafood do you consume per week? (100–150 g of fish, 4–5 pieces or 200 g of seafood)	1 point given if ≥3
11. How many times do you consume commercial (not homemade) pastry such as cookies or cake per week?	1 point given if <2
12. How many times do you consume nuts per week? (1 serving = 30 g)	1 point given if ≥3
13. Do you prefer to eat chicken, turkey or rabbit instead of beef, pork, hamburgers, or sausages?	1 point given: yes
14. How many times per week do you consume boiled vegetables, pasta, rice, or other dishes with a sauce of tomato, garlic, onion, or leeks sautéed in olive oil?	1 point given if ≥2

^1^ 1 tablespoon = 13.5 g. ^2^ fewer than 1 cup (1 cup = 100 mL) of sugar-sweetened beverages/d.

**Table 2:** Anthropometric and biochemical variables in patients with morbid obesity classified according to their adherence to the Mediterranean diet.

Variables	Total Population	Non-adhMD	adhMD	p *
**N (men/women)**	58 (17/41)	50 (15/35)	8 (2/6)	Ns
**Age (years)**	54.1 ± 14.4	53.7 ± 14.3	57.1 ± 15.2	Ns
**Weight (kg)**	111.2 ± 17.4	110.9 ± 17.9	113.2 ± 16.2	Ns
**BMI (kg/m^2^)**	44.1 ± 4.6	44.0 ± 4.7	44.4 ± 4.0	Ns
**Waist (cm)**	126.8 ± 13.7	127.3 ± 13.5	124.1 ± 15.6	Ns
**Hip (cm)**	132.5 ± 11.1	131.4 ± 10.2	137.2 ± 14.7	Ns
**SBP (mmHg)**	135.1 ± 14.7	136.6 ± 16.1	133.8 ± 10.0	Ns
**DBP (mmHg)**	81.4 ± 9.3	82.1 ± 9.2	73.6 ± 9.6	0.04
**Glucose (mg/dL)**	122.0 ± 40.2	124.1 ± 42.5	109.3 ± 17.5	Ns
**Cholesterol (mg/dL)**	195.9 ± 36.7	194.9 ± 36.8	202.0 ± 39.7	Ns
**HDL (mg/dL)**	47.6 ± 11.6	47.1 ± 11.9	50.1 ± 9.8	Ns
**LDL (mg/dL)**	107.0 ± 28.4	106.0 ± 28.1	112.8 ± 31.0	Ns
**Triglycerides (mg/dL)**	143.4 ± 65.9	145.5 ± 69.4	130.4 ± 40.0	Ns
**Insulin (mIU/L)**	19.1 ± 17.5	20.2 ± 18.6	12.2 ± 5.0	Ns
**HOMA-IR**	5.9 ± 8.2	6.9 ± 8.8	3.3 ± 1.6	Ns
**%Patients who met the waist circumference criterion of MS**	100	100	100	Ns
**%Patients who met the triglycerides criterion of MS**	37.5	38.1	25.0	Ns
**%Patients who met the HDL criterion of MS**	60.7	63.8	50.0	Ns
**%Patients who met the hypertension criterion of MS**	72.4	73.5	75.0	Ns
**%Patients who met the glucose or T2DM criterion of MS**	71.9	72.9	62.5	Ns

BMI: body mass index. SBP: systolic blood pressure. DBP: diastolic blood pressure. MS: metabolic syndrome. Results are given as the mean ± standard deviation or as percentage. *p* *: significant differences between adherence and non-adherence to Mediterranean diet. Ns: not significant.

## Data Availability

Data available on request due to ethical and privacy restrictions.
